# Extracellular matrix proteins are time‐dependent and regional‐specific markers in experimental diffuse brain injury

**DOI:** 10.1002/brb3.1767

**Published:** 2020-07-23

**Authors:** Daniel R. Griffiths, Taylor M. Jenkins, Caroline P. Addington, Sarah E. Stabenfeldt, Jonathan Lifshitz

**Affiliations:** ^1^ BARROW Neurological Institute at Phoenix Children’s Hospital Phoenix AZ USA; ^2^ Department of Child Health University of Arizona College of Medicine ‐ Phoenix Phoenix AZ USA; ^3^ School of Biological and Health Systems Engineering, Ira A. Fulton Schools of Engineering Arizona State University Tempe AZ USA; ^4^ Phoenix VA Health Care System Phoenix AZ USA

**Keywords:** extracellular matrix, primary somatosensory barrel cortex, protein, S1BF, traumatic brain injury, ventral posterior medial, ventral posterior medial thalamus

## Abstract

**Introduction:**

The extracellular matrix (ECM) provides structural support for neuronal, glial, and vascular components of the brain, and regulates intercellular signaling required for cellular morphogenesis, differentiation and homeostasis. We hypothesize that the pathophysiology of diffuse brain injury impacts the ECM in a multi‐dimensional way across brain regions and over time, which could facilitate damage and repair processes.

**Methods:**

Experimental diffuse TBI was induced in male Sprague‐Dawley rats (325–375 g) by midline fluid percussion injury (FPI); uninjured sham rats serve as controls. Tissue from the cortex, thalamus, and hippocampus was collected at 15 min, 1, 2, 6, and 18 hr postinjury as well as 1, 3, 7, and 14 days postinjury. All samples were quantified by Western blot for glycoproteins: fibronectin, laminin, reelin, and tenascin‐C. Band intensities were normalized to sham and relative to β‐actin.

**Results:**

In the cortex, fibronectin decreased significantly at 15 min, 1 hr, and 2 hr postinjury, while tenascin‐C decreased significantly at 7 and 14 days postinjury. In the thalamus, reelin decreased significantly at 2 hr, 3 and 14 days postinjury. In the hippocampus, tenascin‐C increased significantly at 15 min and 7 days postinjury.

**Conclusion:**

Acute changes in the levels of these glycoproteins suggest involvement in circuit dismantling, whereas postacute levels may indicate a restorative or regenerative response associated with recovery from TBI.

## Graphical abstract

The extracellular matrix (ECM) provides structural support for neuronal, glial, and vascular components of the brain, and regulates intercellular signaling required for cellular morphogenesis, differentiation, and homeostasis. We hypothesize that the ECM is susceptible to degradation and accumulation of glycoproteins, which can serve as spatial–temporal biomarkers of diffuse traumatic brain injury (TBI).

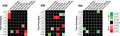

## 1 INTRODUCTION

The central nervous system (CNS) is composed of interacting compartments, including neurons, glia, vasculature, and cerebral spinal fluid. The extracellular matrix (ECM) links these compartments together through ligand–receptor interactions including integrins, cell adhesion molecules, and cell‐surface glycoproteins. The ECM constitutes approximately 20% of the mature brain volume and is comprised of an intricate interconnected matrix of a variety of glycoproteins, proteoglycans, and glycosaminoglycans that stabilize connections between and among CNS compartments (Nicholson & Sykova, 1998). The ECM, as with its interconnections to other CNS compartments, is likely susceptible to the direct mechanical forces of traumatic brain injury (TBI).

In TBI, mechanical forces are applied to the CNS, disrupting the patency of CNS compartments and possibly the integrity of the ECM (George & Geller, 2018). Primary injury is the direct mechanical force to the CNS resulting in tissue damage or destruction, impaired cerebral blood flow, and derailment of cerebral metabolism (Werner & Engelhard, 2007), which can compromise directly the structural integrity of the ECM. Subsequent secondary injury processes include free radical damage, protease activation, and catabolic processes, including break down of the blood–brain barrier and other cellular structures, which may or may not lead to cell death (Werner & Engelhard, 2007). Protease activation can lead to enzymatic degradation of ECM molecules. In this situation, mechanical, pathological, and enzyme activation of ECM molecules could reliably represent the timing and severity of brain injury, if not also relate to longer‐term outcome.

Significant alterations in ECM have been reported in neurodegenerative disease, such as Alzheimer's and Parkinson's Disease (Bonneh‐Barkay & Wiley, 2009; Lu, Takai, Weaver, & Werb, 2011), indicating that the ECM may be involved in the progression of brain injury pathology. It was thought, for example, that senile plaques (SPs) and neurofibrillary tangles (NFTs) were the hallmark pathophysiology of Alzheimer's Disease (AD) (Bonneh‐Barkay & Wiley, 2009). In recent years, ECM components, such as proteoglycans CSPG and HSPG (glypican, syndecans 1–3, and agrin), have been implicated in the early neurodegenerative process (Bonneh‐Barkay & Wiley, 2009). The abundance and early presentation of aberrant ECM in AD support the hypothesis that ECM may be indicative of disease and a viable source of biomarkers in the early pathology of brain injury as well.

A subset of glycoproteins represents the complexity of the ECM with respect to TBI vulnerability, including fibronectin, laminin, reelin, and tenascin‐C.

Fibronectin and laminin are involved in cell adhesion, growth, migration, and differentiation. A focal cortical impact experimental model of TBI increased fibronectin and laminin reactivity in the injury penumbra up to 14 days postinjury compared to uninjured controls (Tate, Tate, & LaPlaca, 2007). Further, macrophages and activated microglia were present predominantly in fibronectin rich tissue, suggesting that fibronectin plays a role in facilitating debris clearing (Tate, Tate, et al., 2007). Furthermore, reactive astrocyte processes were found sheathing laminin‐positive vasculature, suggesting that laminin might play a role in repairing the blood–brain barrier (Tate, Tate, et al., 2007). Thus, fibronectin and laminin may play a role in brain injury repair.

Reelin is an extracellular matrix glycoprotein that plays a role in neuronal migration and positioning during early brain development. In the adult brain, it modulates synaptic plasticity. The ApoE receptors to which reelin binds have been indirectly implicated in memory and neurodegenerative disorders because their ligand, ApoE, is genetically associated with Alzheimer disease (D'Arcangelo, 2005). Thus, reelin may play a role in the progression of brain injury pathology.

In the developing central nervous system, tenascin‐C is involved in regulating the proliferation of both oligodendrocyte precursor cells and astrocytes (Holley, Gveric, Whatmore, & Gutowski, 2005). Expression of tenascin‐C by radial glia precedes the onset of gliogenesis and then drives differentiation of astrocytes (Holley et al., 2005). In the adult brain, tenascin‐C expression is down‐regulated except for the areas that maintain neurogenesis into adulthood and the hypothalamus (Holley et al., 2005). In vitro, tenascin‐C induces a quiescent phenotype in human astrocytes, such that therapeutic strategies to increase tenascin‐C may reduce astrocytic scarring (Holley et al., 2005). Thus, tenascin‐C may serve as a reporter of pathological tissue in the adult brain.

Here, we hypothesize that the pathophysiology of diffuse brain injury impacts the ECM in a multi‐dimensional way across brain regions and over time, which could facilitate damage and repair processes. Using the midline fluid percussion injury model to induce injury, we collected tissue over the time course from 1 hr to 14 days in order to span the initial injury, to circuit dismantling, and the repair of the tissue (Lifshitz et al., 2016). Using Western blots, we quantified the protein levels of fibronectin, laminin, reelin, and tenascin‐C in comparison with uninjured sham values. Primarily, we found that the ECM is susceptible to diffuse TBI.

## 2 METHODS

### 2.1 Midline fluid percussion brain injury

Adult male Sprague‐Dawley rats (355 ± 15 g) were subjected to midline fluid percussion injury (FPI) consistent with methods described previously (Hosseini & Lifshitz, 2009; Lifshitz et al., 2016; Rowe, Griffiths, & Lifshitz, 2016). Briefly, rats were anesthetized with 5% isoflurane in room air and maintained at 2% via nose cone. During surgery, body temperature was maintained with a Deltaphase^®^ isothermal heating pad (Braintree Scientific Inc.). In a head holder assembly (Kopf Instrument), a midline scalp incision exposed the skull. A 4.8‐mm circular craniotomy was performed (centered on the sagittal suture midway between bregma and lambda) without disrupting the underlying dura or superior sagittal sinus. An injury cap was fabricated from the female portion of a Luer‐Loc needle hub, which was cut, beveled, and scored to fit within the craniotomy. A skull screw was secured in a 1‐mm hand‐drilled hole into the right frontal bone. The injury hub was affixed over the craniotomy using cyanoacrylate gel, and methyl‐methacrylate (Hygenic Corp.) was applied around the injury hub and screw. The incision was sutured at the anterior and posterior edges, and topical Lidocaine ointment was applied. Animals were returned to a warmed holding cage and monitored until ambulatory (approximately 60–90 min).

For injury induction, animals were re‐anesthetized with 5% isoflurane at least 60–90 min after surgery. The dura was inspected through the injury‐hub assembly, which was then filled with normal saline and attached to the male end of the fluid percussion device (Custom Design and Fabrication, Virginia Commonwealth University). Animals were randomly assigned to receive a mild to moderate brain injury (*n* = 84; 1.79 ± 0.05 atm) or sham injury (*n* = 12) was administered by releasing (or not releasing) the pendulum onto the fluid‐filled cylinder, as reflexive responses returned. Animals were monitored for the presence of a forearm fencing response and the return of the righting reflex as indicators of injury severity (Hosseini & Lifshitz, 2009). After injury, the injury‐hub assembly was removed en bloc, integrity of the dura was observed, bleeding was controlled with Gelfoam (Pharmacia), and the incision was stapled. Brain‐injured animals had righting reflex recovery times averaging 6:16 ± 0:14 min, and sham‐injured animals recovered within 15 s. After recovery of the righting reflex, animals were placed in a warmed holding cage before being returned to the housing room. Experiments were conducted in accordance with NIH and institutional guidelines concerning the care and use of laboratory animals. Adequate measures were taken to minimize pain or discomfort. In the conduct of this study, four rats died from pulmonary edema prior to their predetermined endpoint. Final animal numbers are *n* = 8 for each brain injury time point and *n* = 6 for sham animals at 1 and 7 days postsurgery.

### 2.2 Tissue collection and processing

At predetermined time points, each animal was given a lethal dose of sodium pentobarbital 200 mg/kg (Euthasol^®^, i.p.). Animals were transcardially perfused with ice‐cold phosphate‐buffered saline (PBS) for 1–2 min. The brain was rapidly removed and rinsed with ice‐cold PBS. Bilateral tissue biopsies (2 mm diameter) from the primary somatosensory barrel cortex (S1BF), ventral posterior medial (VPM) nucleus of the thalamus, and hippocampus were collected from 2 mm thick coronal sections off a chilled rat brain matrix. Tissue samples were flash‐frozen and stored at 221280°C until protein was extracted for Western blot analysis.

Total protein was extracted from S1BF, VPM, and hippocampus biopsies. Tissues were homogenized in 250 μl of ice‐cold extraction buffer (pH 8.0) containing 0.24 M Tris, 0.74 M NaCl, 100 μl TritonX100 with a protease inhibitor cocktail (complete, Roche Diagnostics; #11836153001). Tissue biopsies were homogenized with the Precellys^®^24 machine (Bertin Technologies) for 2 × 20 s. Samples were then centrifuged at 3,000 *g* for 15 min and the supernatant collected for analysis. Protein concentrations were determined using the bicinchoninic acid assay (BCA) using manufacturer's instructions (Pierce).

### 2.3 Western blots

NuPAGE^®^ LDS Sample Buffer (4X; Life Technologies; NP0007) and NuPAGE^®^ Sample Reducing Agent (10X; Life Technologies; NP0004) were added to the protein samples and then boiled for 10 min at 70°C. Protein extracts (20 μg) were then electrophoresed in a 3%–8% Tris‐Acetate Midi gel (Life Technologies; WG1603BOX) and transferred to nitrocellulose membranes with iBlot^®^ Transfer Stack (Life Technologies; IB3010‐01) using iBlot^®^ Blotting System (Invitrogen) for 9:30 min. Blots were blocked in 1x PBS with 3% milk and 0.1% Tween‐20 buffer for 1 hr at room temperature. Blots were incubated with primary antibodies to fibronectin (abcam, AB2413, 1:1,000), pan laminin (Sigma‐Aldrich, L9393, 1:1,000), reelin (Millipore, MAB5364, 1:500), or tenascin‐C (abcam, ab108930, 1:500) overnight at 4°C. After rinsing the blot 3 times with 1X PBS + 0.1% Tween20 over 30 min, blots were incubated with secondary antibody (LiCor^®^ IRDye, 1:5,000–1:10,000) for 1 hr. Blots were washed three times with 1X PBS + 0.1% Tween20 over 30 min. Bands were visualized using the LiCor^®^ ODYSSEY^®^ Classic and analyzed by Image Studio™ 4.0 software (LiCor). As a loading control, β‐actin (Sigma‐Aldrich, A5441, 1:15,000) protein levels were measured. Samples were randomized and run in triplicate. Sham samples were used as loading controls and run on all gels. Samples were excluded if they were >2.5 standard deviations from the mean. Densitometry was determined for fibronectin, laminin, reelin, or tenascin‐C band intensity relative to β‐actin expression; cleavage products were quantified for reelin and tenascin‐C. Anti‐Fibronectin antibody (ab2413) detects a single band with a predicted molecular weight of 262 kDa and an observed molecular weight of 285 kDa by ABCAM. We observed and analyzed a single band at 270 kDa, similar to ABCAM and other users. Laminin is comprised of three chains, designated A (molecular weight 400 kDa), B1 (molecular weight 210 kDa), and B2 (molecular weight 200 kDa) (Kleinman, Luckenbill‐Edds, Cannon, & Sephel, 1987). We observed and analyzed a single band at 415 kDa. Reelin (MAB5364) has a predicted molecular weight of ~388 kDa. We observed and analyzed multiple reelin bands at 210, 290, and 415 kDa. Tenascin‐C is a large extracellular matrix molecule comprised of individual polypeptides with molecular weights ranging from ~180 to ~300 kDa (Jones & Jones, 2000a, 2000b). We observed and analyzed multiple bands for tenascin‐c at 250 and 290 kDa. β‐actin (A5441) has a predicticted molecular weight of 42 kDa. We observed and analyzed a single band at 45 kDa. Replicates of the protein of interest to β‐actin levels were averaged to obtain the expression per animal per region; individual animal values were averaged within a group.

**Figure 1:**
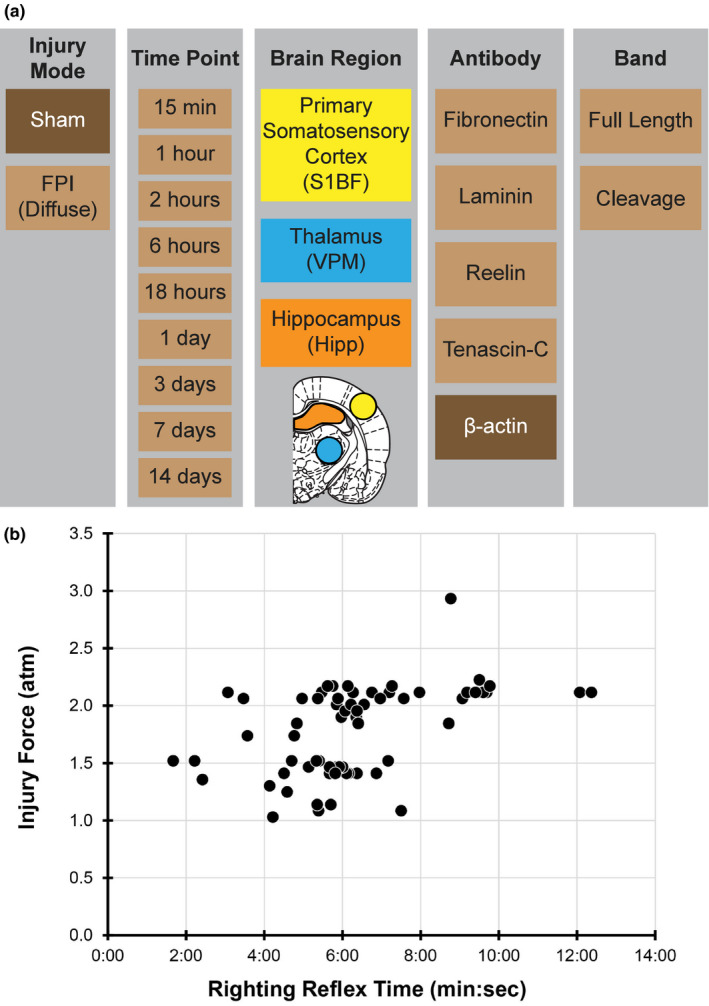
(a) Tabular depiction of the study design, including injury mode, time points postinjury, brain regions, extracellular matrix antibodies, and the bands of interest for quantification. Uninjured sham and β‐actin are colored to indicate their role as a control. (b) Scatter plot of injury force (atm) from the fluid percussion injury (FPI) device versus the righting reflex physiological measure of injury severity (min:s). The graph conveys the range of injury forces included in the study, which is heterogeneous as in the clinic

A graphical representation of the study design accounting for the pertinent independent and dependent variables is presented in Figure 1.

### 2.4 Statistical analysis

A range of injury pressures (1.03–2.93 atm) were included in the study to better represent the heterogeneity of injury severity reported in the clinic. We did not observe an effect of injury pressure in any outcome and elected not to stratify the injury group by injury severity. Heat maps were generated to aid in the graphical visualization and identification of regional and temporal changes in protein levels. S1BF, VPM, and hippocampus heat maps were generated from statistical results comparing protein expression against the normalized uninjured sham value of 1.0, relative to β‐actin expression. Optical densities of bands at indicated molecular weights (kDa) were divided by β‐actin expression and normalized to the mean expression for uninjured sham. For expression levels >2.0 or <0.5, a one tailed *t* test was run against the predicted value of 1.0. Adjusted *p*‐values for the nine comparisons over time postinjury are plotted against color scale indicating the degree of significance (*p* value), where green indicates increased expression and red indicates decreased expression compared to sham. The heat map visualization identified significant changes within regions and time points in protein levels. The identified regions and time points were regraphed. Significant changes in protein levels are indicated on the graphs, even if they did not meet the initial criteria >2.0 or <0.5 threshold of detection for the heat map. Full Western blots are shown in Figure S1 for each antibody at representative time points postinjury.

## 3 RESULTS

### 3.1 Heat maps identify significant differences in ECM molecules over time postinjury across brain regions

**Figure 2:**
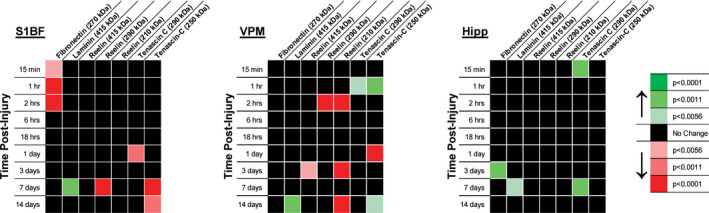
Primary somatosensory barrel field (S1BF), ventral posterior medial (VPM) nucleus of the thalamus, and hippocampus (Hipp) heat maps were generated from statistical results comparing protein expression against a normalized uninjured sham value of 1.0, relative to β‐actin expression. Optical densities of bands at indicated molecular weights (kDa) were divided by β‐actin expression and normalized to the mean expression in uninjured sham. When expression levels were > 2.0 or < 0.5, a one tailed *t* test was run against the predicted value of 1.0. Adjusted *p*‐values for the nine comparisons over time postinjury are plotted against colors indicating the degree of significance, where green indicates increased expression and red indicates decreased expression compared to sham

Heat maps were developed to visualize data for initial analysis of trends over time and between ECM molecules (Figure 2). For S1BF, with the exception of laminin, ECM molecules decreased at acute (fibronectin) and chronic (reelin and tenascin‐C) time points postinjury. Fibronectin was decreased 52% at 15 min (*t*(8) = 3.59, *p* < .0056), decreased 54% at 1 hr (*t*(8) = 8.73, *p* < .0001), and decreased 57% at 2 hr (*t*(8) = 7.29, *p* < .0001) postinjury. Laminin was increased 414% 7 days (*t*(8) = 6.70, *p* < .0011) postinjury. The 290 kDa reelin band was decreased 53% at 7 days (*t*(7) = 7.94, *p* < .0001) postinjury. The 290 kDa tenascin‐C band was decreased 60% 1 day (*t*(8) = 5.11, *p* < .0011) postinjury. The 250 kDa tenascin‐C band was decreased 89% at 7 days (*t*(8) = 95.85, *p* < .0001) and decreased 69% at 14 days (*t*(5) = 6.99, *p* < .0011) postinjury. The heat map did not identify any significant changes in the S1BF for the 415 kDa or 210 kDa reelin bands.

For VPM, reelin breakdown products decrease at acute and chronic time points postinjury, while tenascin‐C product may have a biphasic response. Laminin was increased 239% at 14 days (*t*(7) = 6.08, *p* < .0011) postinjury. The 415 kDa reelin band was decreased 61% at 3 days (*t*(6) = 4.78, *p* < .0056) postinjury. The 290 kDa reelin band was decreased 61% at 2 hr (*t*(8) = 14.93, *p* < .0001) postinjury. The 210 kDa reelin band was decreased 53% at 2 hr (*t*(8) = 13.65, *p* < .0001), decreased 51% at 3 days (*t*(8) = 8.92, *p* < .0001) and decreased 52% at 14 days (*t*(7) = 10.62, *p* < .0001) postinjury. The 290 kDa tenascin‐C band was increased 210% at 1 hr (*t*(8) = 4.10, *p* < .0056) postinjury. The 250 kDa tenascin‐C band was increased 233% at 1 hr (*t*(8) = 6.15, *p* < .0011), decreased 67% at 1 day (*t*(8) = 8.79, *p* < .0001), and increased 252% at 14 days (*t*(7) = 3.84, *p* < .0056) postinjury. No significant changes were identified in the VPM for fibronectin.

For hippocampus, ECM molecules were increased, except for reelin. Fibronectin was increased 281% at 3 days (*t*(8) = 2.53, *p* < .0011) postinjury. Laminin was increased 208% at 7 days (*t*(8) = 3.78, *p* < .0056) postinjury. The 290 kDa tenascin‐C band was increased 202% at 15 min (*t*(8) = 5.00, *p* < .0011) and increased 237% at 7 days (*t*(8) = 5.66, *p* < .0011) postinjury. The heat map did not reveal any significant changes in the hippocampus for the 415, 290, or 290 kDa reelin bands or the 250 kDa tenascin‐C band.

### 3.2 Temporal expression profiles of ECM molecules

**Figure 3:**
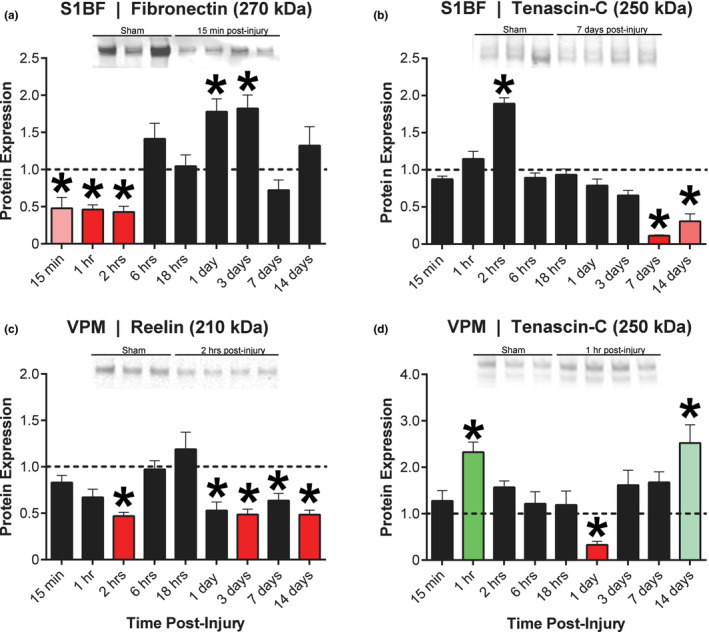
Complete temporal profiles for four combinations of brain region and protein bands identified in the heat maps. (a, b) Primary somatosensory barrel field (S1BF) showed an early reduction in fibronectin (270 kDa) and late reduction in tenascin‐C (250 kDa) expression compared to normalized sham value of 1.0 (horizontal dashed line). (c, d) Ventral posterior medial (VPM) nucleus of the thalamus showed sustained reduction in reelin (210 kDa) and bimodal increased expression in tenascin‐C (250 kDa), with a decreased expression at 1 day postinjury. No additional analysis was conducted for the hippocampus. *, *p* value as indicated in Figure 2, compared to normalized sham value

Based on trends revealed by the heat maps, specific temporal expression profiles were extracted for detailed analysis (Figure 3). The temporal profile in the S1BF revealed significant changes in fibronectin compared to sham (Figure 3a). Fibronectin was decreased 52% at 15 min (*t*(8) = 3.59, *p* < .0056), decreased 54% at 1 hr (*t*(8) = 8.73, *p* < .0001) and 57% at 2 hr (*t*(8) = 7.29, *p* < .0001) postinjury compared to sham. Fibronectin returned to sham values by 18 hr postinjury. Fibronectin was increased 178% at 1 day (*t*(8) = 4.50, *p* < .0056) and increased 182% at 3 days (*t*(8) = 4.52, *p* < .0056) postinjury compared to sham. The increase in fibronectin at 1 and 3 days postinjury was not highlighted by the heat map because the change was below the 200% threshold of detection. Fibronectin returned to sham values by 14 days postinjury. Overall, fibronectin was decrease initially after injury before a sustained increase in S1BF from 1 to 3 days postinjury.

The temporal profile in the S1BF revealed significant changes to the 250 kDa tenascin‐C band compared to sham (Figure 3b). For the first hours postinjury, tenascin‐C does not differ from sham values. Tenascin‐C was increased 189% (below the heat map threshold) at 2 hr (*t*(8) = 11.2, *p* < .0001) postinjury compared to sham. Tenascin‐C returned to sham values by 18 hr postinjury. The 250 kDa tenascin‐C band was decreased 89% at 7 days (*t*(8) = 95.85, *p* < .0001) and decreased 69% at 14 days (*t*(5) = 6.99, *p* < .0011) postinjury compared to sham. Overall, tenascin‐C has an acute increase followed by a chronic decrease postinjury compared to sham.

The temporal profile in the VPM revealed significant changes to the 210 kDa reelin band compared to sham (Figure 3c). Reelin was decreased to 53% of sham values over the first 2 hr (*t*(8) = 13.65, *p* < .0001) postinjury compared to sham. Reelin returned to sham values by 6 hr postinjury. Reelin was decreased 47% at 1 day (*t*(8) = 5.13, *p* < .0011), decreased 51% at 3 days (*t*(8) = 2.25, *p* < .0001), decreased 37% at 7 days (*t*(6) = 4.60, *p* < .0056) and 52% at 14 days (*t*(7) = 2.05, *p* < .0001) postinjury compared to sham. The decrease in reelin was highlighted by the heat map at 3 days and 14 days postinjury, but not at 1 day and 7 days postinjury because the change was below the 50% threshold of detection. Overall, the 210 kDa reelin band is decreased after TBI compared to sham.

The temporal profile in the VPM revealed significant changes to the 250 kDa band of tenascin‐C compared to sham (Figure 3d). Tenascin‐C was increased 233% over the first hour (*t*(8) = 6.15, *p* < .0011) postinjury compared to sham. Tenascin‐C returned to sham values 6 hr postinjury. Tenascin‐C was decreased 67% at 1 day (*t*(8) = 8.79, *p* < .0001) postinjury compared to sham. Tenascin‐C returned to sham values by 3 days postinjury. Tenascin‐C was increased 252% at 14 days (*t*(7) = 3.84, *p* < .0056) postinjury compared to sham. Overall, tenascin‐C has a biphasic response, with an early and late increases over sham values after TBI.

### 3.3 Regional expression profiles of ECM molecules

**Figure 4:**
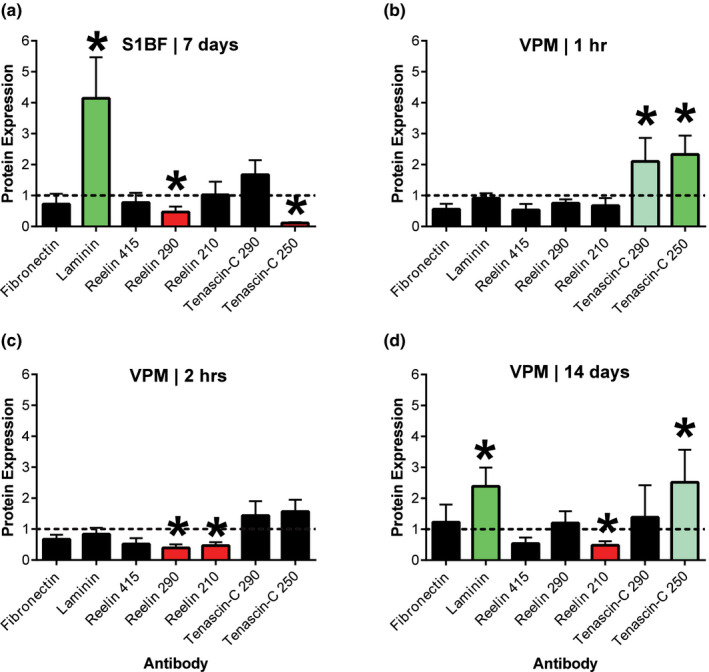
Complete spatial profiles for four combinations of brain region and time point identified by the heat maps. (a) Primary somatosensory barrel field (S1BF) at 7 days postinjury showed an early increase in laminin and reductions in reelin (290 kDa) and tenascin‐C (250 kDa) expression compared to normalized sham value of 1.0 (horizontal dashed line). (b) Ventral posterior medial (VPM) nucleus of the thalamus at 1 hr postinjury showed increased expression of both tenascin‐C bands. (c) VPM at 2 hr postinjury showed a reduction in expression of both reelin bands. (d) VPM at 14 days postinjury showed increased expression of laminin and tenascin‐C (250 kDa), with a reduction in reelin (210 kDa). No additional analysis was conducted for the hippocampus. *, *p* value as indicated in Figure 2, compared to normalized sham value

Based on trends revealed by the heat maps, specific regional expression profiles for all ECM molecules were extracted for detailed analysis (Figure 4). The regional profile for the S1BF at 7 days postinjury revealed significant changes in laminin, reelin, and tenascin‐C compared to sham (Figure 4a). Laminin was increased 314% (*t*(8) = 6.70, *p* < .0001), the 290 kDa reelin band was decreased 53% (*t*(7) = 7.94, *p* < .0001), and the 250 kDa tenascin‐C band was decreased 89% (*t*(8) = 95.85, *p* < .0001) compared to sham. Overall, the ~ fourfold increase in laminin is the largest significant result.

The regional profile for the VPM at 1 hr postinjury revealed significant changes to tenascin‐C compared to sham (Figure 4b). The 290 kDa tenascin‐C band was increased 210% (*t*(8) = 4.10, *p* < .0056), and the 250 kDa tenascin‐C band was increased 233% (*t*(8) = 6.15, *p* < .0011) compared to sham.

The regional profile for the VPM at 2 hr postinjury revealed significant changes to reelin compared to sham (Figure 4c). The 290 kDa reelin band was decreased 61% (*t*(8) = 14.93, *p* < .0001), and the 210 kDa reelin band was decreased 53% (*t*(8) = 13.65, *p* < .0001) compared to sham.

The regional profile for the VPM at 14 days postinjury revealed significant changes to laminin, reelin, and tenascin‐C compared to sham (Figure 4d). The laminin band was increased 239% (*t*(7) = 6.08, *p* < .0011), the 210 kDa reelin band was decreased 52% (*t*(7) = 10.62, *p* < .0001), and the 250 kDa tenascin‐C band was increased 252% (*t*(7) = 3.84, *p* < .0056) compared to sham.

## 4 DISCUSSION

The ECM is a component of the neurovascular unit that provides structural support for neuronal, glial, and vascular components of the brain, while regulating intercellular signaling required for cellular morphogenesis, differentiation, and homeostasis (George & Geller, 2018). The ECM is remodeled constantly during normal brain function, and amplified in recovery, repair, and regeneration of the damaged central nervous system. In the context of TBI, mechanical forces of injury and enzymatic activity can target the ECM to aid or hinder both injury and repair processes (Bonneh‐Barkay & Wiley, 2009). Here, the results indicate that cortex, thalamus, and hippocampus are vulnerable to experimental diffuse TBI with regard to the temporal expression of ECM molecules. In the cortex, fibronectin was decreased significantly at 15 min, 1 and 2 hr postinjury, while tenascin‐C was decreased significantly at 7 and 14 days postinjury. In the thalamus, reelin was decreased significantly at 2 hr, 3 and 14 days postinjury. In the hippocampus, tenascin‐C was increased significantly at 15 min and 7 days postinjury. These results indicate that the ECM is dynamically remodeled during the pathophysiology of diffuse TBI, whereby changes in these glycoprotein levels at acute time points may represent circuit dismantling, and then circuit reorganization at chronic time points.

Fibronectin is an ECM molecule that is found in high concentrations during development, but is largely absent in healthy adult brain tissue (Tate, Garcia, Garcia, & LaPlaca, 2007). Fibronectin can be either a soluble form produced by hepatocytes or an insoluble form produced locally by a variety of cells. Soluble fibronectin may act as an opsonin, helping macrophages identify and remove dead tissue and cellular debris (Lu et al., 2011; Tate, Garcia, et al., 2007). Insoluble fibronectin is an ECM molecule associated with cellular adhesion, spreading, proliferation, and migration (To & Midwood, 2011). Brain injury increases fibronectin levels, predominantly the soluble form (Tate, Garcia, et al., 2007). Over two hours after diffuse TBI, fibronectin levels decreased in the S1BF cortex, and then a sustained increase over 3 days postinjury. The acute decrease in fibronectin observed in the cortex may represent clearance of fibronectin‐coated debris by macrophages, including fibronectin protein, from regions of mechanical impact. The sustained increase in fibronectin may represent plasma fibronectin leakage into the parenchyma through a compromised blood–brain barrier. One may propose that fibronectin levels are re‐established once the blood–brain barrier seals several days postinjury (Schmidt & Grady, 1993). The delayed significant increases in cortex and hippocampus fibronectin may represent protracted pathophysiology and repair. In the chronic phase of repair, ECM may need to be dissolved by matrix metalloproteinases to facilitate neuronal outgrowth as part of regeneration and repair (Abdul‐Muneer, Pfister, Haorah, & Chandra, 2016).

Laminin is a major component of the basal lamina and regulates cell adhesion, migration, differentiation, and proliferation (Hamill, Kligys, Hopkinson, & Jones, 2009). Beyond a potential role in debris clearance, laminin could repair the blood–brain barrier by way of basal lamina and astrocyte associations (George & Geller, 2018). Laminin levels were increased in a delayed manner (7 or 14 days postinjury) across all three brain regions, which suggest a role in repair and restoration of the injured brain, possibly at the interface of the blood–brain barrier.

Reelin is a key factor in the regulation of neuronal migration and layer formation in the developing brain. Reelin regulates cell growth, maturation, and synaptic activity in the adult brain (Gabriella D'Arcangelo, 2014). Reelin interacts with two receptors to regulate synaptic plasticity, apolipoprotein E receptor 2 (ApoER2) and very‐low‐density‐lipoprotein receptor (VLDLR). ApoER2 is important for proper migration of neurons, while VLDLR may act as a stop signal for migrating neurons (Hack et al., 2007). Reelin decreased after TBI in the cortex and thalamus. The absence of reelin opens the window for thrombospondin‐1 to interact with VLDLR and ApoER2 (Blake et al., 2008). Thrombospondin activates the α2δ‐1 subunit on voltage‐gated calcium channels to promote synaptogenesis (Risher & Eroglu, 2012). Work in our laboratory continues to evaluate thrombospondin mediated plasticity after TBI, showing increases by three days after fluid percussion injury in the rat followed by an increase in synaptic markers (GAP43 and PICK‐1) (Ogle et al., 2016). Decreased reelin expression levels after TBI may promote plasticity through thrombospondin interactions with VLDLR and ApoER2.

Tenascin‐C plays a role in the migration of immature neurons, with trophic and repulsive properties. Expression is highest during embryonic development, tissue repair, and in conditions of chronic inflammation (Midwood & Orend, 2009). In adulthood, tenascin‐C is likely down‐regulated, except in areas that maintain neurogenesis throughout life, such as the hippocampus (Holley et al., 2005). The genetic knock‐down of tenascin‐C showed its importance in the formation of contextual memories and synaptic plasticity in the hippocampus (Strekalova et al., 2002). We interpret the increase in hippocampal tenascin‐C at 7 days postinjury to reinforce the framework of circuit reorganization. Alternatively, tenascin‐C can impede terminal sprouting and act as a stop signal for injury‐induced plasticity (Niquet, Jorquera, Faissner, Ben‐Ari, & Represa, 1995), whereby aberrant plasticity would be regulated in the recovery of the injured brain (Thomas, Hinzman, Gerhardt, & Lifshitz, 2012).

Part of TBI pathophysiology is inflammation, protease activation, and ECM degradation, which together define the injured brain. Protease activation includes calpains, caspases, and matrix metalloproteinases (MMP), which can go on to degrade the ECM (Knoblach & Faden, 2005). Calpains and caspases may interact with laminin, contributing to breakdown of the blood–brain barrier (BBB) (Knoblach & Faden, 2005). MMP‐9 can digest the fibronectin and laminin proteins in the vascular basal lamina. In this way, protease activity after TBI can disrupt the BBB and promote vasogenic edema (Wang et al., 2000). Protease inhibitors have been used to reduce or prevent disruption of the BBB after TBI, which preserved BBB integrity at least temporarily (Knoblach & Faden, 2005). Therefore, the ECM represents a downstream target of pathophysiological processes that may contribute to degeneration or restoration of the injured brain.

Experimental models of TBI do not replicate the human condition perfectly, whereby models represent a fraction of the heterogeneous clinical conditions. Yet, these results extend the investigations on the response of the extracellular matrix to diffuse brain injury in the cortex, thalamus, and hippocampus. In the progression of TBI from the initiating event into the disease process, affected brain regions necessarily undergo pathology, degeneration, repair, and regeneration (Lifshitz et al., 2016). In this progression, neural circuits are dismantled, followed by circuit reorganization. In the acute phase, cellular debris accumulates, including components of the ECM. In the latter phases, new or existing cells (neurons and glia) need to repair or rebuild the damaged or missing circuits. And so, the ECM either hinders or promotes repair and regeneration, depending on the spatial–temporal expression profile of ECM molecules. If the ECM remains unaffected by injury, then regenerating neurons must grow through a mature brain; if the ECM is proteolytically processed to expose cryptic sites, then neurites extend through a debris field of attractive and repulsive epitopes; if the ECM is remodeled prior to neural regeneration, perhaps the conditions are optimal for repair of the injured nervous system.

If ECM protein levels are markedly elevated after brain injury, with the ECM as a critical component of the basal lamina of the blood–brain barrier, then the potential exists for ECM molecules to leak into systemic circulation. As a potential TBI biomarker (Mondello et al., 2011), peripheral ECM molecules could inform TBI severity or predict functional outcome. To date, accepted biomarkers originate intracellularly from glia or neurons. For example, S100β is a calcium‐binding protein found in astrocytes (Unden, Ingebrigtsen, & Romner, 2013) and neuron‐specific enolase is a glycolytic enzyme in the neuronal cytoplasm (Di Battista, Rhind, & Baker, 2013). To reach peripheral circulation, intracellular proteins must traverse the cell membrane, the ECM, and then the blood–brain barrier. On the other hand, ECM molecules may be cleared more readily into peripheral circulation. Further investigation into ECM fragment levels in systemic circulation is encouraged. Although blood was collected in the present study, technical challenges with Western blots or absence of meaningful change precluded presentation (data not shown). Yet, the high abundance of ECM proteins in all organs encourage future investigation into a brain‐specific cleavage sites of ECM molecules as a TBI biomarker.

Limitations of this study include the selected model, gross dissection of brain regions, and the focus on a subset of all ECM molecules. In the diffuse TBI model, the absence of frank tissue degeneration and restricted blood–brain barrier permeability may have tempered the magnitude or duration of ECM molecule expression (Bharadwaj, Lifshitz, Adelson, Kodibagkar, & Stabenfeldt, 2016; Bharadwaj et al., 2018). A focal brain injury, such as controlled cortical impact, may demonstrate an alternate utility of ECM molecules as biomarkers or targets after TBI. Also, tissues were collected from cortical and subcortical brain regions, but not separated by parenchyma, vasculature, or blood–brain barrier, which could distinguish specific compartments where ECM molecule expression contributes to the pathophysiology and recovery from TBI. Last, the Western blot approach to quantify ECM molecule expression suffers a selection bias of the ECM molecules of interest due to finite sample sizes; untargeted proteomics may provide a broader assessment of the ECM response to TBI.

## 5 CONCLUSION

In this study, we found that the protein levels of fibronectin, laminin, reelin, and tenascin‐C are altered over an acute (15 min) to chronic (14 days) timeframe that involves circuit dismantling and reorganization across the cortex, thalamus, and hippocampus. Acute changes in ECM proteins may represent consequences of the physical trauma to the brain and the associated neuroinflammation. Subacute changes may represent the reparative and restorative processes. We have shown that the ECM undergoes multi‐dimensional changes following TBI that are likely involved in circuit dismantling and repair. Further investigation is needed to understand how specific ECM proteins and their splice variants contribute to TBI pathophysiology and recovery.

## CONFLICT OF INTEREST

All authors declare that there are no actual or potential conflicts of interest including any financial, personal, or other relationships with other people or organizations that could inappropriately influence this work.

## AUTHOR CONTRIBUTION

Sarah Stabenfeldt and Jonathan Lifshitz designed the study. Daniel Griffiths, Taylor Jenkins, and Caroline Addington collected tissue, processed samples, and analyzed data for publication. Sarah Stabenfeldt and Jonathan Lifshitz oversaw data analysis and interpretation. Daniel Griffiths and Taylor Jenkins wrote the manuscript with the help of Sarah Stabenfeldt and Jonathan Lifshitz.

### Peer Review

The peer review history for this article is available at https://publons.com/publon/10.1002/brb3.1767.

## Supporting information

FigS1

## Data Availability

The data that support the findings of this study are available from the corresponding author upon reasonable request.

## References

[brb31767-bib-0001] Abdul‐Muneer, P. M. , Pfister, B. J. , Haorah, J. , & Chandra, N. (2016). Role of matrix metalloproteinases in the pathogenesis of traumatic brain injury. Molecular Neurobiology, 53(9), 6106–6123. 10.1007/s12035-015-9520-8 26541883 PMC9470225

[brb31767-bib-0002] Bharadwaj, V. N. , Lifshitz, J. , Adelson, P. D. , Kodibagkar, V. D. , & Stabenfeldt, S. E. (2016). Temporal assessment of nanoparticle accumulation after experimental brain injury: Effect of particle size. Scientific Reports, 6, 29988 10.1038/srep29988 27444615 PMC4957235

[brb31767-bib-0003] Bharadwaj, V. N. , Rowe, R. K. , Harrison, J. , Wu, C. , Anderson, T. R. , Lifshitz, J. , … Stabenfeldt, S. E. (2018). Blood‐brainbarrier disruption dictates nanoparticle accumulation following experimental brain injury. Nanomedicine, 14(7), 2155–2166. 10.1016/j.nano.2018.06.004 29933022 PMC6177306

[brb31767-bib-0004] Blake, S. M. , Strasser, V. , Andrade, N. , Duit, S. , Hofbauer, R. , Schneider, W. J. , & Nimpf, J. (2008). Thrombospondin‐1 binds to ApoER2 and VLDL receptor and functions in postnatal neuronal migration. EMBO Journal, 27(22), 3069–3080. 10.1038/emboj.2008.223 18946489 PMC2585172

[brb31767-bib-0005] Bonneh‐Barkay, D. , & Wiley, C. A. (2009). Brain extracellular matrix in neurodegeneration. Brain Pathology, 19(4), 573–585. 10.1111/j.1750-3639.2008.00195.x 18662234 PMC2742568

[brb31767-bib-0006] D'Arcangelo, G. (2005). Apoer2: A reelin receptor to remember. Neuron, 47(4), 471–473. 10.1016/j.neuron.2005.08.001 16102527

[brb31767-bib-0007] D'Arcangelo, G. (2014). Reelin in the years: Controlling Neuronal migration and maturation in the mammalian brain. Advances in Neuroscience, 2014, 1–19. 10.1155/2014/597395

[brb31767-bib-0008] Di Battista, A. P. , Rhind, S. G. , & Baker, A. J. (2013). Application of blood‐based biomarkers in human mild traumatic brain injury. Frontiers in Neurology, 4, 44 10.3389/fneur.2013.00044 23641234 PMC3640204

[brb31767-bib-0009] George, N. , & Geller, H. M. (2018). Extracellular matrix and traumatic brain injury. Journal of Neuroscience Research, 96(4), 573–588. 10.1002/jnr.24151 29344975 PMC5803383

[brb31767-bib-0010] Hack, I. , Hellwig, S. , Junghans, D. , Brunne, B. , Bock, H. H. , Zhao, S. , & Frotscher, M. (2007). Divergent roles of ApoER2 and Vldlr in the migration of cortical neurons. Development, 134(21), 3883–3891. 10.1242/dev.005447 17913789

[brb31767-bib-0011] Hamill, K. J. , Kligys, K. , Hopkinson, S. B. , & Jones, J. C. (2009). Laminin deposition in the extracellular matrix: A complex picture emerges. Journal of Cell Science, 122(Pt 24), 4409–4417. 10.1242/jcs.041095 19955338 PMC2787456

[brb31767-bib-0012] Holley, J. E. , Gveric, D. , Whatmore, J. L. , & Gutowski, N. J. (2005). Tenascin C induces a quiescent phenotype in cultured adult human astrocytes. Glia, 52(1), 53–58. 10.1002/glia.20231 15892123

[brb31767-bib-0013] Hosseini, A. H. , & Lifshitz, J. (2009). Brain injury forces of moderate magnitude elicit the fencing response. Medicine and Science in Sports and Exercise, 41(9), 1687–1697. 10.1249/MSS.0b013e31819fcd1b 19657303 PMC11421656

[brb31767-bib-0014] Jones, F. S. , & Jones, P. L. (2000). The tenascin family of ECM glycoproteins: Structure, function, and regulation during embryonic development and tissue remodeling. Developmental Dynamics, 218(2), 235–259. 10.1002/(sici)1097-0177(200006)218:2<235:aid-dvdy2>3.0.co;2-g 10842355

[brb31767-bib-0015] Jones, P. L. , & Jones, F. S. (2000). Tenascin‐C in development and disease: Gene regulation and cell function. Matrix Biology, 19(7), 581–596. 10.1016/s0945-053x(00)00106-2 11102748

[brb31767-bib-0016] Kleinman, H. , Luckenbill‐Edds, L. , Cannon, F. , & Sephel, G. (1987). Use of extracellular matrix components for cell culture. Analytical Biochemistry, 166(1), 1–13. 10.1016/0003-2697(87)90538-0 3314585

[brb31767-bib-0017] Knoblach, S. M. , & Faden, A. I. (2005). Proteases in traumatic brain injury In Lendeckel U. & Hooper N. M. (Eds.), Proteases in the brain, Proteases In Biology and Disease, Vol. 3 (pp. 79–108). Boston, MA: Springer.

[brb31767-bib-0018] Lifshitz, J. , Rowe, R. K. , Griffiths, D. R. , Evilsizor, M. N. , Thomas, T. C. , Adelson, P. D. , & McIntosh, T. K. (2016). Clinical relevance of midline fluid percussion brain injury: Acute deficits, chronic morbidities and the utility of biomarkers. Brain Injury, 30(11), 1293–1301. 10.1080/02699052.2016.1193628 27712117 PMC5303557

[brb31767-bib-0019] Lu, P. , Takai, K. , Weaver, V. M. , & Werb, Z. (2011). Extracellular matrix degradation and remodeling in development and disease. Cold Spring Harbor Perspectives in Biology, 3(12), a005058 10.1101/cshperspect.a005058 21917992 PMC3225943

[brb31767-bib-0020] Midwood, K. S. , & Orend, G. (2009). The role of tenascin‐C in tissue injury and tumorigenesis. Journal of Cell Communication and Signaling, 3(3–4), 287–310. 10.1007/s12079-009-0075-1 19838819 PMC2778592

[brb31767-bib-0021] Mondello, S. , Muller, U. , Jeromin, A. , Streeter, J. , Hayes, R. L. , & Wang, K. K. (2011). Blood‐based diagnostics of traumatic brain injuries. Expert Review of Molecular Diagnostics, 11(1), 65–78. 10.1586/erm.10.104 21171922 PMC3063529

[brb31767-bib-0022] Nicholson, C. , & Sykova, E. (1998). Extracellular space structure revealed by diffusion analysis. Trends in Neurosciences, 21(5), 207–215. 10.1016/S0166-2236(98)01261-2 9610885

[brb31767-bib-0023] Niquet, J. , Jorquera, I. , Faissner, A. , Ben‐Ari, Y. , & Represa, A. (1995). Gliosis and axonal sprouting in the hippocampus of epileptic rats are associated with an increase of tenascin‐C immunoreactivity. Journal of Neurocytology, 24(8), 611–624. 10.1007/BF01257376 7595669

[brb31767-bib-0024] Ogle, S. , Law, L. , Johnson, S. , Adelson, P. D. , Lifshitz, J. , & Thomas, T. C. (2016). Re‐expression of thrombospondins is concomitant with increased synaptic markers after diffuse traumatic brain injury. Journal of Neurotrauma, 33(13), A76–A77.

[brb31767-bib-0025] Risher, W. C. , & Eroglu, C. (2012). Thrombospondins as key regulators of synaptogenesis in the central nervous system. Matrix Biology, 31(3), 170–177. 10.1016/j.matbio.2012.01.004 22285841 PMC3961754

[brb31767-bib-0026] Rowe, R. K. , Griffiths, D. R. , & Lifshitz, J. (2016). Midline (Central) fluid percussion model of traumatic brain injury. Methods in Molecular Biology, 1462, 211–230. 10.1007/978-1-4939-3816-2_13 27604721

[brb31767-bib-0027] Schmidt, R. H. , & Grady, M. S. (1993). Regional patterns of blood‐brain barrier breakdown following central and lateral fluid percussion injury in rodents. Journal of Neurotrauma, 10(4), 415–430. 10.1089/neu.1993.10.415 8145265

[brb31767-bib-0028] Strekalova, T. , Sun, M. , Sibbe, M. , Evers, M. , Dityatev, A. , Gass, P. , & Schachner, M. (2002). Fibronectin domains of extracellular matrix molecule tenascin‐C modulate hippocampal learning and synaptic plasticity. Molecular and Cellular Neurosciences, 21(1), 173–187. 10.1006/mcne.2002.1172 12359159

[brb31767-bib-0029] Tate, C. C. , Garcia, A. J. , & LaPlaca, M. C. (2007). Plasma fibronectin is neuroprotective following traumatic brain injury. Experimental Neurology, 207(1), 13–22. 10.1016/j.expneurol.2007.05.008 17599836

[brb31767-bib-0030] Tate, C. C. , Tate, M. C. , & LaPlaca, M. C. (2007). Fibronectin and laminin increase in the mouse brain after controlled cortical impact injury. Journal of Neurotrauma, 24(1), 226–230. 10.1089/neu.2006.0043 17263686

[brb31767-bib-0031] Thomas, T. C. , Hinzman, J. M. , Gerhardt, G. A. , & Lifshitz, J. (2012). Hypersensitive glutamate signaling correlates with the development of late‐onset behavioral morbidity in diffuse brain‐injured circuitry. Journal of Neurotrauma, 29(2), 187–200. 10.1089/neu.2011.2091 21939393 PMC3261793

[brb31767-bib-0032] To, W. S. , & Midwood, K. S. (2011). Plasma and cellular fibronectin: Distinct and independent functions during tissue repair. Fibrogenesis Tissue Repair, 4, 21 10.1186/1755-1536-4-21 21923916 PMC3182887

[brb31767-bib-0033] Unden, J. , Ingebrigtsen, T. , & Romner, B. (2013). Scandinavian guidelines for initial management of minimal, mild and moderate head injuries in adults: An evidence and consensus‐based update. BMC Medicine, 11, 50 10.1186/1741-7015-11-50 23432764 PMC3621842

[brb31767-bib-0034] Wang, X. , Jung, J. C. , Asahi, M. , Chwang, W. , Russo, L. , Moskowitz, M. A. , … Lo, E. H. (2000). Effects of matrix metalloproteinase‐9 gene knock‐out on morphological and motor outcomes after traumatic brain injury. Journal of Neuroscience, 20(18), 7037–7042. 10.1523/jneurosci.20-18-07037.2000 10995849 PMC6772824

[brb31767-bib-0035] Werner, C. , & Engelhard, K. (2007). Pathophysiology of traumatic brain injury. British Journal of Anaesthesia, 99(1), 4–9. 10.1093/bja/aem131 17573392

