# Supplementary figures and images for: Extracellular matrix proteins are time‐dependent and regional‐specific markers in experimental diffuse brain injury
Source: Brain Behav. 2020 Jul 23;10(9):e01767. doi: 10.1002/brb3.1767 (PMC7507085; doi:10.1002/brb3.1767)

# Example Western Blots

Hippocampus - 15 min post-injury

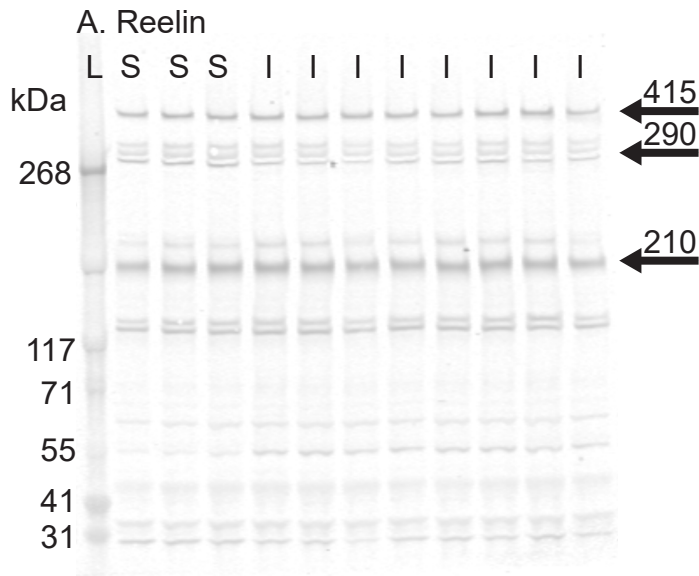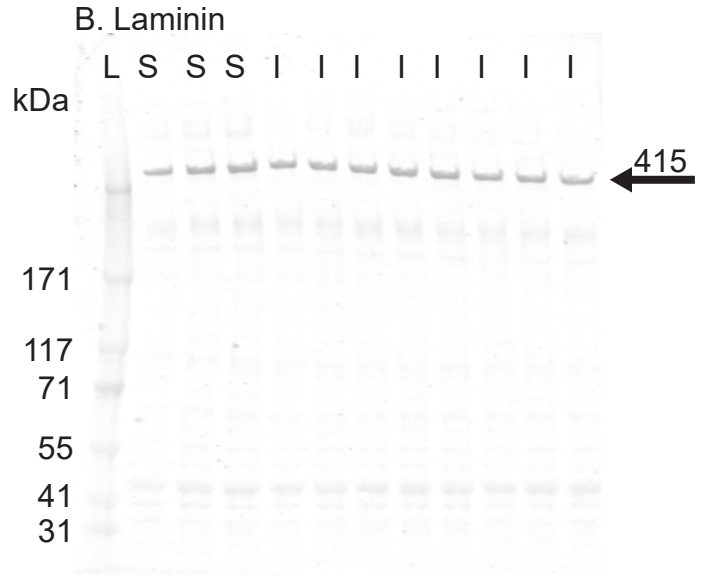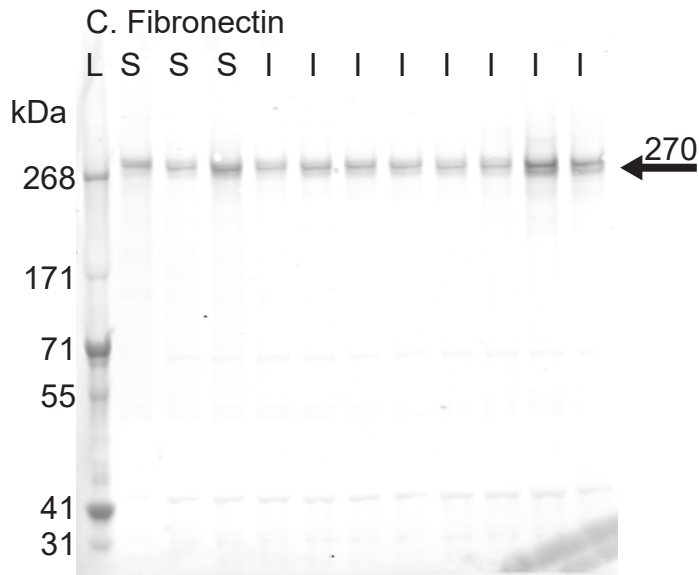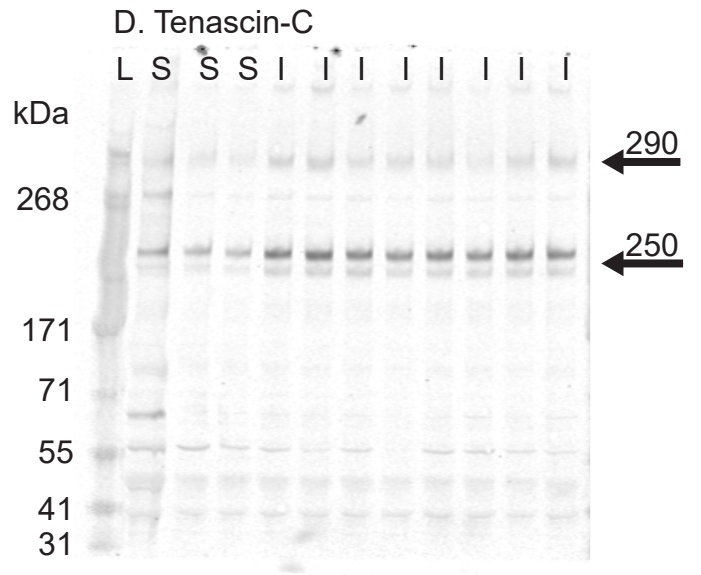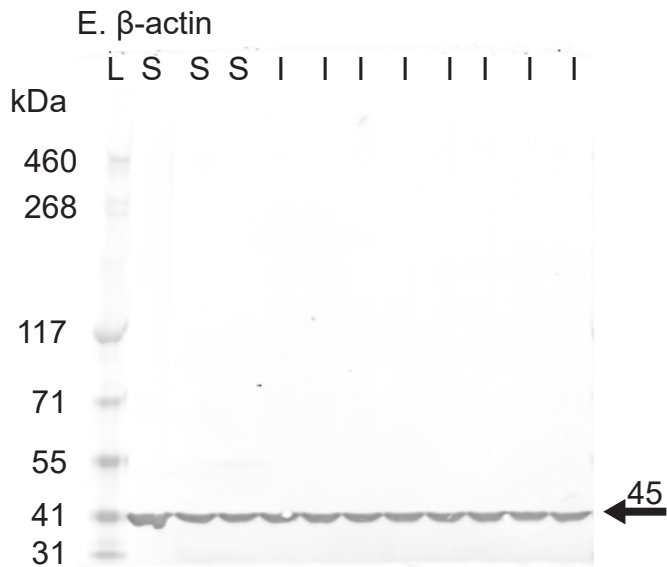

Supplement: Supplementary file 1 — FigS1 [file BRB3-10-e01767-s001.pdf]
